# Mechanically Recycled PLA Films Reinforced with Rice Husk and Carbonized Rice Husk Particles

**DOI:** 10.3390/polym18080982

**Published:** 2026-04-17

**Authors:** Sergio Gonzalez-Serrud, Ana Cristina González-Valoys, Marina P. Arrieta

**Affiliations:** 1 Departamento de Ciencias e Ingeniería de los Materiales, Facultad de Ingeniería Mecánica, Universidad Tecnológica de Panamá, Panama City 0819-07289, Panama; 2 Grupo de Investigación en Geoquímica Aplicada y Sostenibilidad (GeoAS), Universidad Tecnológica de Panamá, Panama City 0819-07289, Panama; ana.gonzalez1@utp.ac.pa; 3 Departamento de Ingeniería Química Industrial y del Medio Ambiente, Escuela Técnica Superior de Ingenieros Industriales, Universidad Politécnica de Madrid, 28006 Madrid, Spain; 4 Facultad de Ingeniería Civil, Universidad Tecnológica de Panamá, Panama City 0819-07289, Panama; 5 Centro de Estudios Multidisciplinarios en Ciencias, Ingeniería y Tecnología (CEMCIT-AIP), Panama City 0819-07289, Panama; 6 Sistema Nacional de Investigación-Secretaria Nacional de Ciencia, Tecnología e Innovación (SNI-SENACYT), Clayton Ciudad del Saber Edif. 205, Panama City 0816-02852, Panama; 7 Grupo de Polímeros, Caracterización y Aplicaciones (POLCA), Universidad Politécnica de Madrid, 28006 Madrid, Spain; 8 Centro de Investigación en Materiales Estructurales (CIME), Universidad Politécnica de Madrid, C/Profesor Aranguren 3, 28040 Madrid, Spain

**Keywords:** mechanically reprocessed PLA, rice husk waste, biochar, biocomposites

## Abstract

This study investigates the development of mechanically reprocessed poly(lactic acid) (rPLA) films reinforced with rice husk (RH) and rice husk biochar (RHB) to evaluate their processing behavior, key functional properties, and disintegration under composting conditions. rPLA was produced from PLA through an additional processing cycle to simulate the valorization of industrial PLA waste, while composites containing 1 and 3 wt.% RH or RHB 500 µm sized particles were manufactured by melt extrusion followed by a compression molding process. Reprocessing increased the melt flow index and decreased intrinsic viscosity and viscosimetric molecular weight, evidencing the occurrence of chain scission during mechanical reprocessing. The addition of RH slightly restricted melt flow and promoted higher surface hydrophilicity, whereas RHB showed a filler-loading-dependent effect on melt flow and increased surface hydrophobicity at low content, consistent with its carbonized and less polar nature. Both RH and RHB promote a nucleating effect, with increased crystallinity in RHB-containing films, and tensile tests showing that filler incorporation mainly reduced ductility compared with unfilled rPLA, while stiffness and strength was maintained or exhibited more moderate variations. Despite these contrasting trends in surface properties and thermo-mechanical performance, all formulations achieved complete disintegration within 21 days under composting conditions at laboratory scale level. Overall, RH and RHB provide a viable route to valorize agro-industrial residues in rPLA films and to tune structure–property relationships within the circular economy framework.

## 1. Introduction

The increasing environmental burden of persistent petroleum-derived plastics, together with the need to reduce dependence on fossil resources, has accelerated the development of polymeric materials compatible with circular economy strategies. Within this context, polylactic acid (PLA) stands out as a bio-based and biodegradable polymer produced from renewable feedstocks such as corn starch or sugarcane, and it is biocompatible and biodegradable being currently widely adopted in biomedical applications, packaging, disposable products, etc. [1,2,3]. More than 40% of the global plastic production is used for packaging manufacturing and the compostable character of PLA has generated great interest in packaging applications. However, the broader use of PLA in single-use products is still limited due to its moderate thermal resistance and limited mechanical robustness, particularly under demanding service conditions [1]. Several research efforts have been focused on improving PLA mechanical resistance mainly focused on the development of composites and nanocomposites [4,5].

To reduce both the fossil dependance of plastics and the accumulation in the environment, the recently adopted European Packaging and Packaging Waste Regulation (PPWR) EU 2025/40 establishes mandatory minimum recycled content targets for plastic packaging to be achieved by 2030 [6]. Although PLA is inherently compostable, it is not exempt from these regulatory commitments. Therefore, the development of effective recycling strategies for PLA is essential to ensure its compliance with forthcoming recycled content requirements. For instance, contact-sensitive packaging made from plastics other than PET must contain a minimum of 10% post-consumer recycled material [7].

Beyond material selection, the mechanical integrity of polymeric components is strongly governed by the processing–structure–property relationship. In PLA, processing history and end-of-life pathways can significantly modify chain architecture and microstructure, ultimately affecting stiffness, strength, and failure behavior. Mechanical recycling, a key route in circularity, can promote thermo-mechanical degradation and chain scission, which may reduce molecular weight and alter rheological behavior [8]. These changes affect melt processability and may compromise final mechanical performance. Therefore, strategies that restore or improve the mechanical response of mechanically recycled PLA (rPLA) while preserving sustainability benefits remain of high interest [9].

One interesting valorization pathway is the reprocessing of industrial PLA waste such as defective plastics parts discarded from the production line, production scraps, burrs, etc., as they are of a well-known origin and composition, ensuring consistency in PLA grade [10]. Moreover, as they have not entered conventional post-consumer waste streams, they typically do not require complex sorting and intensive washing steps, unlike post-consumer PLA waste [10,11], thereby facilitating a homogeneous and food-grade material as well as a more efficient and economically viable recycling process [10].

A promising approach to increase the mechanical properties of the materials is the development of PLA-based biocomposites reinforced with agricultural by-products, which can reduce the amount of PLA in the formulation and valorize waste streams, lessening the environmental footprint [12]. In this context, rice is one of the most widely produced and consumed staple crops worldwide, and rice husk (RH) is an abundant lignocellulosic by-product generated by the rice milling industry with low cost and wide availability in rice producing countries. Meanwhile, rice husk biochar (RHB) is its carbonized counterpart with distinct surface chemistry and thermal stability [1,3]. The biochar derived from carbonized rice husk (RHB) has been recognized for its ability to improve soil quality, primarily due to its content of essential nutrients such as nitrogen. This element plays a crucial role in soil fertility, facilitating nutrient availability to plants and enhancing the soil’s water retention capacity. These properties contribute to the development of a more efficient and sustainable agricultural ecosystem [13].

The incorporation of RH and RHB to PLA can modify its melt flow and crystallization behavior and can also influence mechanical behavior and failure mechanisms through changes in interfacial adhesion finding a value addition to rise waste as a secondary source of material. From a structural integrity perspective, it is important to determine whether RH and RHB increase stiffness at the expense of ductility or enable a better balance between stiffness, strength, and strain-to-failure in PLA films [12].

Although PLA composites reinforced with natural fillers as well as biochar have been widely reported, most studies have focused on virgin PLA matrices or on only one rice-husk-derived reinforcement considered separately [14,15,16]. In contrast, the novelty of the present work lies in the combined evaluation of mechanically reprocessed PLA, together with the direct comparison between untreated rice husk (RH) and rice husk biochar (RHB) 500 µm particles within the same film-processing route and at the same filler loading. This approach makes it possible to differentiate how the nature of the rice-husk-derived reinforcement affects melt processability, molecular degradation, thermo-mechanical response, surface-related properties, and disintegration under controlled composting conditions. Therefore, this study specifically contributes to understanding how agro-industrial residues can be integrated into reprocessed PLA films to tune performance while maintaining a circular end-of-life perspective.

For the best of our knowledge, mechanically recycled PLA coming from a closed loop, such as those from the industrial sector, have not been previously blended with rice husk and/or rice husk biochar. Consequently, this study shows the effect of addition of 500 µm sized particles of RH and RHB into mechanically recycled PLA films, focusing on how filler type, natural RH or carbonized RHB influences processing-related parameters and the resulting thermo-mechanical performance of the mechanically recycled composites. For that, PLA was reprocessed to simulate industrial PLA waste valorization throughout a mechanical recycling process by a second melt extrusion step (rPLA) following a previous work [10]. The RH particles were sieved to obtain a particle size smaller than 500 µm, while RH were further converted into biochar (RHB) by pyrolysis at 450 °C. The obtained rPLA was further loaded with both untreated rice husk (RH) and biochar (RHB) in 1 and 3 wt.%. The obtained composite films were fully characterized by assessing the intrinsic viscosity, chemical structure, microstructure, tensile properties, and thermal properties, together with water-barrier-related properties and disintegration under controlled composting conditions at laboratory scale level. Overall, this work aims to clarify processing–structure relationships in rPLA biocomposite films reinforced with agro-industrial residues from the rice field, supporting the design of sustainable materials within circular economy frameworks.

## 2. Materials and Methods

### 2.1. Materials

Poly(lactic acid) (PLA) pellets (Ingeo™ 2003D, M_n_ ≈ 70,000 Da, 2 wt.% D-isomer) were supplied by NatureWorks LLC (Minnetonka, MN, USA). PLA utilized in this study includes both PLA and reprocessed PLA (rPLA) to simulate the valorization of industrial PLA waste. rPLA represents PLA that has undergone an additional processing cycle, simulating the valorization of defective plastic parts from the industry following a previously developed procedure [10].

The rice husk was supplied by Cooperativa Avance R.L., Los Olivos, Los Santos, Panama. The untreated rice husk (RH) particles were filtered through a 500-micron mesh. The rice husk (RH) was converted into biochar (RHB) by pyrolysis in a fixed-bed reactor at 450 °C (heating rate: 10 °C/min) under an inert N_2_ atmosphere (12 mL/min), in the absence of O_2_. This optimum temperature was chosen based on previous studies indicating that biochar produced at this rate has a balanced cost–benefit [9].

The films’ formulations were fabricated using 1 or 3 wt.% content of reinforcing fillers HB or RHB to assess the impact of bio-based particles on the properties of rPLA. Each blend was processed via melt extrusion followed by compression molding in a hot press to form films, facilitating initial evaluations at low reinforcement concentrations of 1 wt.% and 3 wt.%. These filler contents allowed for the observation of significant changes in mechanical, thermal, and biodegradability properties without compromising the material’s processability [11,17].

#### 2.1.1. Material Processing

**Figure 1:**
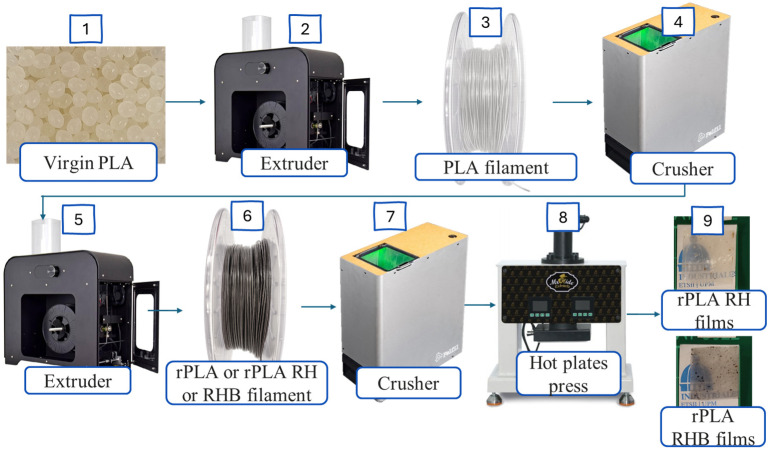
Methodology used to manufacture PLA, rPLA films.

Figure 1 shows schematically the manufacturing of PLA films with the corresponding additives starting from rPLA pellet production to film manufacturing.

Figure 1 shows the processing methodology used to manufacture the PLA-based films evaluated in this work. Initially, virgin poly(lactic acid) (PLA) pellets were dried overnight at 60 °C in a vacuum oven (J.P. Selecta, Barcelona, Spain) to minimize hydrolytic degradation during melt processing. In the first stage, the dried PLA pellets were processed in a 3DEVO Composer 350 extruder (3Devo, Utrecht, The Netherlands) to obtain PLA filament.

The temperature profile from hopper to nozzle was set at 170, 185, 190, and 170 °C, respectively, and the screw speed was fixed at 4.5 rpm. The resulting PLA filament was subsequently ground in a Felfil Plastic Shredder+ Model 500 (Felfil, Turin, Italy) to obtain PLA particles suitable for reprocessing and homogeneous blending with the fillers. In the second stage, these particles were re-extruded in the same 3DEVO Composer 350 extruder (3Devo, Utrecht, The Netherlands) to simulate the mechanical recycling of industrial PLA waste and to produce either reprocessed PLA (rPLA) filament or composite filaments containing rice husk (RH) or rice husk biochar (RHB).

For the composite systems, the mechanically recycled PLA flakes were previously dry and mixed with 1 and 3 wt.% of RH or RHB in a sealed glass jar to promote a uniform distribution of the filler before the second blending extrusion step. The resulting formulations were designated as rPLA-1%RH, rPLA-3%RH, rPLA-1%RHB, and rPLA-3%RHB.

After reprocessing, the obtained filaments were ground again in the Felfil Plastic Shredder+ Model 500 and then processed into film by compression molding in a hot-plate press, Mr. Hide Extracts WTRP-10T Rosin press (Tarragona, Spain), to obtain the final films, including neat rPLA, rPLA/RH, and rPLA/RHB films. In this way, the methodology integrated drying, extrusion, grinding, re-extrusion, dry blending, and compression molding to produce both unfilled and filled films under a comparable processing route.

#### 2.1.2. Film Manufacturing

Previously dried rPLA pellets (60 °C in a vacuum oven (J.P. Selecta oven, Barcelona, Spain) were compressed in a hot-plate press to obtain the films (Mr. Hide Extracts WTRP-10T Rosin press, Tarragona, Spain). A specifically designed mold for films with dimensions of 15 × 15 cm^2^ was utilized. Initially, 2 g of each composite formulation was placed in the mold at 180 °C under atmospheric pressure conditions. This setup was maintained for 2 min to allow for the complete melting of the material. Subsequently, a sequential compression cycle was implemented to optimize the quality of the films and to eliminate any air bubbles potentially trapped within the material. The films were processed at a constant temperature of 180 °C, under the following pressure cycle: Initially, the films were kept at atmospheric pressure for one minute, followed by the application of 3 MPa for another minute. The pressure was then increased to 5 MPa for an additional minute, and finally, 10 MPa was applied for two more minutes to ensure the removal of air bubbles from the film matrix. After the compression molding process, the films were cooled to room temperature inside the plate molds under atmospheric pressure with an ice pack in contact with it to ensure rapid cooling to obtain transparent films [18].

### 2.2. Melt Flow Index (MFI)

To evaluate the melt processability of PLA, rPLA, rPLA-RH, and rPLA-RHB pellets, the melt flow index (MFI) was determined using a Metrotec MFI-100 device (Techlab Systems, Lezo, Spain). The measurements were carried out at a constant temperature of 190 °C under a 2.16 kg load. For each material, six consecutive tests were performed, and each measurement was recorded over a period of 15 s.

### 2.3. Viscosity Molecular Weight

The intrinsic viscosity ([η]) of all the PLA-based bionanocomposite films was assessed by measuring the capillary viscosity using a Ubbelohde viscometer (type C), in compliance with UNE-EN ISO 1628-1:2021 standard [19]. Each sample was diluted in chloroform (Sigma-Aldrich, Burlington, MA, USA, 99% purity) and the viscosity measurements were performed in a water bath maintained at a constant temperature of 25 °C, using at least five different concentrations for each sample. Subsequently, the molecular weight of viscosity (M_v_) was calculated using the Mark–Houvink equation—[η] = K × (M_v_)^a^—where K and a are constants valued at 1.53 × 10^−2^ and 0.759 respectively, specifically for PLA [20].

### 2.4. Mechanical Properties

Tensile testing was conducted at ambient temperature using a Shimadzu AGS-X-100N universal testing machine, which is equipped with a 100 N load cell (Shimadzu, Kyoto, Japan). The procedure adhered to the standardized protocol UNE-EN ISO 527-3:2018 [21]. Tests were carried out on rectangular samples measuring 5 mm by 30 mm, with an initial separation of 20 mm between grips and a crosshead speed set at 5 mm per minute. From the stress–strain curves obtained, average values for percentage strain at break (ɛ_b_), elastic modulus (E_t_), and tensile strength (σ_t_) were calculated, based on six measurements for each type of biocomposite.

### 2.5. Differential Scanning Calorimetry (DSC)

The thermal transitions of PLA, rPLA, rPLA 1%RH, rPLA 3%RH, rPLA 1%RHB and rPLA 3%RHB produced in this study were evaluated using differential scanning calorimetry (DSC). Thermograms were obtained in a SETLINE DSC from SETARAM (Caluire, France) through a three-step temperature cycle, which included an initial heating from 25 °C to 200 °C to remove thermal history, followed by cooling to 0 °C, and a final heating to 240 °C. The tests were conducted at 10 °C/min in a nitrogen atmosphere at a flow rate of 30 mL/min. Samples weighing between 5 and 8 mg were analyzed in sealed 40 µL aluminum crucibles. Crystallinity (X_c_) was calculated based on melting and cold crystallization enthalpies (ΔH_m_ and ΔH_cc_, respectively), with the reference melting enthalpy for fully crystalline PLA (ΔH_0m_) set at 93 J/g [22].

(1) $$X_{C}\left(\%\right)=\left[\frac{\Delta H_{m}-\Delta H_{cc}}{\Delta H_{0m}\cdot \left(1-w\right)}\right]\cdot 100$$

### 2.6. Thermogravimetric Analysis (TGA)

Dynamic thermogravimetric analyses were done in a TGA 2050 Thermogravimetric Analyzer, SETARAM (Caluire, France). One sample for each composite was placed in the analysis cell. Approximately 10 mg of samples were placed in a platinum pan and subjected to a heating ramp from 40 to 800 °C, at a rate of 10 °C/min, all within a nitrogen atmosphere. The sample was subjected to a constant temperature increase, while continuously measuring the weight loss or gain of the sample.

### 2.7. Attenuated Total Reflectance-Fourier Transform Infrared Spectroscopy (ATR-FTIR)

Fourier Transform Infrared Spectroscopy examination was conducted utilizing a 4X ATR-FTIR spectrometer manufactured by Jasco Corporation, headquartered in Hachioji, Tokyo, Japan. Absorbance measurements were performed across a wavelength span ranging from 4000 cm^−1^ to 400 cm^−1^, employing 24 scan repetitions and a resolution of 4 cm^−1^.

### 2.8. Static Contact Angle Measurements

The surface wettability of the films was evaluated through static water contact angle (WCA) measurements using a standard goniometer (Ossila BV, Leiden, The Netherlands) equipped with a camera and Ossila Software version 4.1.4. Drops of approximately 10 µL of distilled water were placed onto the film surfaces using a syringe, and around ten contact angle measurements were taken for each sample, with the films positioned randomly.

### 2.9. Water Absorption

The water absorption characteristics of the developed PLA bionanocomposite films were evaluated following the guidelines set forth in UNE-EN ISO 62:2008 [23]. For this evaluation, square specimens measuring 15 × 15 mm^2^ were submerged in deionized water for 60 days at room temperature of 23 ± 1 °C. Throughout this testing period, the samples were removed from the water on a weekly basis, gently dried using an absorbent towel, and weighed using a AX125D analytical balance from Nänikon, Uster, Switzerland, which offers an accuracy of 1 × 10^−5^ g. After each weighing, the samples were re-immersed in the deionized water. To ensure the reliability of the results, all tests were conducted in triplicate [22]. The water absorption rate was calculated using a specific Formula (2):

(2) $$\%\;\;water\;absorption=\;\frac{Mm-\;Md}{Md}\;\times \;100\%$$

where Mm is the mass of the sample after exposure to moisture and Md is the mass of the dry sample.

### 2.10. Water Vapor Transmission Rate

The water vapor transmission rate (WVTR) of the bio-based film composites was assessed using gravimetric methods, where silica gel served as the desiccant. Each film, covering an area of 10 cm^2^, was placed over permeability cups containing 2 g of silica gel. These setups were then stored in a desiccator maintained at 23 ± 1 °C with a relative humidity of approximately 90%, using a saturated potassium nitrate (KNO_3_) solution. The weight of each film formulation was recorded hourly over a six-hour period. The WVTR, expressed in grams per day per square centimeter, was determined using the equation where mt represents the weight of the cup at time t, m0 is the initial weight, and A is the film’s exposed area. The results were adjusted to reflect a standard thickness of 100 micrometers [24].

(3) $$WVTR=\frac{240\times \left(m_{t}-m_{0}\right)}{A\times t}.$$

### 2.11. Field Emission Scanning Electron Microscopy (FESEM)

The cross-sectional morphology of the cryogenically fractured surfaces of each film sample was examined using field emission scanning electron microscopy (FESEM). Prior to analysis, the samples were frozen in liquid nitrogen (N_2_) and cut to reveal the cross-section, followed by sputter-coating with a gold–palladium alloy.

### 2.12. Disintegration Under Composting Conditions

The decomposition of mats under composting conditions was evaluated at a laboratory scale in accordance with UNE-EN ISO 20200:2024 standard [25]. Film squares measuring 15 mm by 15 mm were placed in mesh textile bags for exposure to the compost medium and to further facilitate the subsequent recovery [18]. Samples were then buried at depths of 4 to 6 cm within perforated plastic containers filled with a synthetic wet waste mixture. This mixture consisted of 10% compost (Mantillo, Granada, Spain), 30% rabbit food, 10% starch, 5% sugar, 1% urea, 4% corn oil, 40% sawdust, and was hydrated to approximately 50% water content by weight. The film samples were maintained under aerobic conditions at a temperature of 58 °C and retrieved for analysis after 1, 4, 7, 9, 11, 14, 18 and 21 days to monitor the progress of disintegration. Photographs were taken of each sample upon retrieval to qualitatively assess the extent of physical breakdown over time.

### 2.13. Statistical Analysis

A completely randomized experimental design was adopted for this study. Statistical analyses were performed using Python 3.13.12. Analysis of variance (ANOVA) was used to evaluate the data, and Fisher’s least significant difference (LSD) test was applied to determine differences among samples. Statistical significance was established at *p* < 0.05.

## 3. Results and Discussions

### 3.1. Melt Flow Index (MFI)

**Figure 2:**
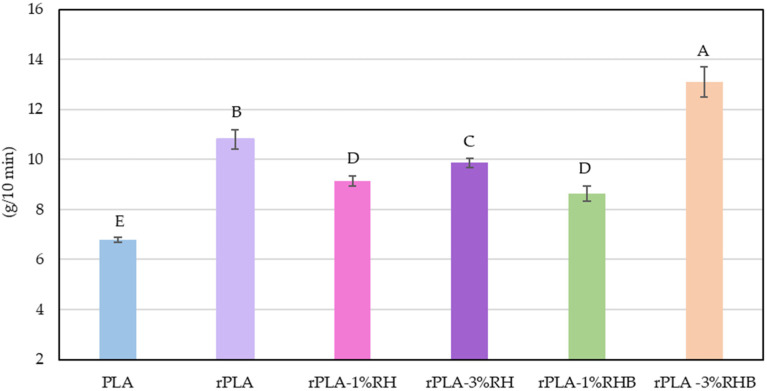
Determination of the melt flow index (190 °C, 2.16 kg) of PLA, rPLA, and rPLA composite pellets in bulk. ^A–E^ Different letters indicate statistically significant differences between formulations *p* < 0.05.

The melt flow index (MFI) results are presented in Figure 2. The commercial PLA pellet exhibited an MFI of 6.8 g/10 min, in line with values reported for virgin PLA grades [10]. In contrast, the reprocessed PLA (rPLA) displayed a marked increase in MFI (10.8 g/10 min), consistent with chain scission effects induced by thermal and mechanical stresses during extrusion. Similar values were obtained by Agüero et al. after subjecting PLA to one and two reprocessing cycles [26]. This increase indicates a reduction in molecular weight and melt viscosity, phenomena widely reported in PLA subjected to multiple processing cycles [26,27,28].

When RH was introduced into the rPLA matrix, a partial decrease in MFI was observed: 9.15 g/10 min for 1 wt.% RH and 9.86 g/10 min for 3 wt.% RH. The presence of lignocellulosic particles may contribute to increased resistance to flow, possibly due to polymer-filler interfacial friction or fiber entanglement with polymer chains, which offsets part of the fluidity gained from reprocessing [29]. Similar values to that for rPLA-1%RH of MFI were obtained for rPLA-3%RH. The nonlinear behavior with RH content suggests complex interactions between filler dispersion, fiber orientation, and rheological behavior at low filler loadings [30].

Interestingly, samples reinforced with carbonized RH (RHB) exhibited different trends. At 1 wt.% RHB, the MFI further decreased to 8.64 g/10 min, reinforcing the hypothesis that RHB introduces a more pronounced barrier effect during flow, due to its more rigid, thermally stable, and porous structure [9]. However, at 3 wt.% RHB, the MFI significantly increased to 13.1 g/10 min, surpassing even the rPLA MFI value. This sharp rise may be attributed to synergistic effects between high filler content and matrix degradation, where RHB particles, being less polar, interact less with the PLA matrix. The low interfacial adhesion promotes polymer chain slippage and reduces entanglement density, especially in matrices already weakened by thermal history. Such behavior has been previously reported in biochar-reinforced PLA systems where excessive filler loading promotes flow due to poor interfacial adhesion [31]. Additionally, biochar derived from rice husk can contain residual inorganic species (e.g., silica and mineral ash) as well as oxygen-containing surface functionalities that may act as catalytic sites under melt-processing conditions, promoting chain scission reactions in PLA during processing.

Overall, the MFI results reflect a delicate balance between matrix degradation due to reprocessing and the rheological influence of RH or RHB as fillers. While reprocessing reduces molecular weight and enhances flow, the incorporation of RH tends to moderately restrict melt mobility, whereas RHB exhibits a dual effect depending on its concentration. These findings are crucial for optimizing the extrusion conditions of rPLA-based biocomposites, especially when targeting applications requiring specific melt flow behavior.

### 3.2. Viscosity Molecular Weight

**Figure 3:**
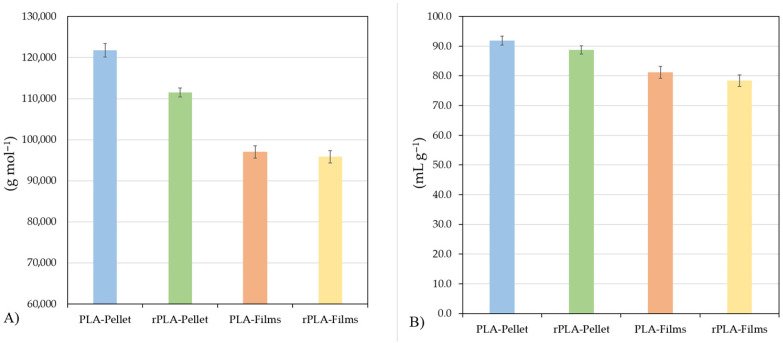
(**A**) Viscosity molecular weight and (**B**) intrinsic viscosity of PLA and rPLA-based pellets and films.

Figure 3 presents the results from the viscosity molecular weight analysis of various PLA films and pellets.

The evaluation of the viscosity molecular weight (M_v_) and intrinsic viscosity ([η]) of PLA and rPLA, both in pellet and film forms, reveals a clear reduction in these parameters after the material undergoes thermal processing stages (Figure 2). Specifically, the viscosity molecular weight of neat PLA pellets was 121,771 ± 1600 g·mol^−1^, with an intrinsic viscosity of 91.8 ± 1.5 mL·g^−1^. In contrast, rPLA pellets exhibited lower values, with an M_v_ of 111,530 ± 1100 g·mol^−1^ and an intrinsic viscosity of 88.7 ± 1.4 mL·g^−1^, corresponding to a reduction of approximately 8.4% and 3.4%, respectively, when compared to PLA pellets. This initial decrease is indicative of the thermo-mechanical degradation suffered by the material during reprocessing cycles. The transformation of PLA into rPLA involves an additional extrusion cycle, where high temperatures and shear stress promote chain scission reactions, reducing the molecular weight of the polymer. These results are consistent with previous studies, which have reported that reprocessing of PLA leads to a gradual decrease in molecular weight due to the breakage of ester bonds and the formation of shorter polymer chains [32]. Further reductions in both M_v_ and intrinsic viscosity were observed in PLA and rPLA films. PLA films exhibited an M_v_ of 96,996 ± 1500 g·mol^−1^ and an intrinsic viscosity of 81.1 ± 2 mL·g^−1^, representing a decrease of 20.3% and 11.6%, respectively, relative to PLA pellets. Similarly, rPLA films showed an M_v_ of 95,830 ± 1500 g·mol^−1^ and an intrinsic viscosity of 78.4 ± 2 mL·g^−1^, evidencing a reduction of 14.1% and 11.7%, respectively, when compared to rPLA pellets. Similar findings were observed by Aldhafeeri et al., (2022) [27].

These results confirm that the extrusion and compression molding processes used for film production further accelerate the degradation of the polymer chains. The combined effect of high temperatures, shear forces, and oxygen exposure during melt processing promotes chain scission and a reduction in molecular weight, which directly influences the intrinsic viscosity of the material. This behavior is characteristic of PLA-based materials, which are susceptible to hydrolytic and thermal degradation during processing [10].

The progressive decrease on the viscosity molecular weight and intrinsic viscosity not only impacts the rheological properties of the material but also has significant implications for the mechanical and barrier performance of the films. Lower molecular weight results in reduced chain entanglement and cohesive forces within the matrix, potentially compromising tensile strength, elongation at break, and resistance to water vapor permeation [26].

From a sustainability standpoint, these findings highlight the need to control processing parameters and evaluate potential solutions, such as the incorporation of chain extenders, antioxidants, or optimized processing conditions, to mitigate the degradation of rPLA during recycling cycles. The observed reductions in molecular parameters will serve as a critical reference point to further analyze the influence of rice husk and carbonized rice husk incorporation on the physicochemical integrity and performance of the developed biocomposite films [33].

### 3.3. Attenuated Total Reflectance-Fourier Transform Infrared Spectroscopy (ATR-FTIR)

**Figure 4:**
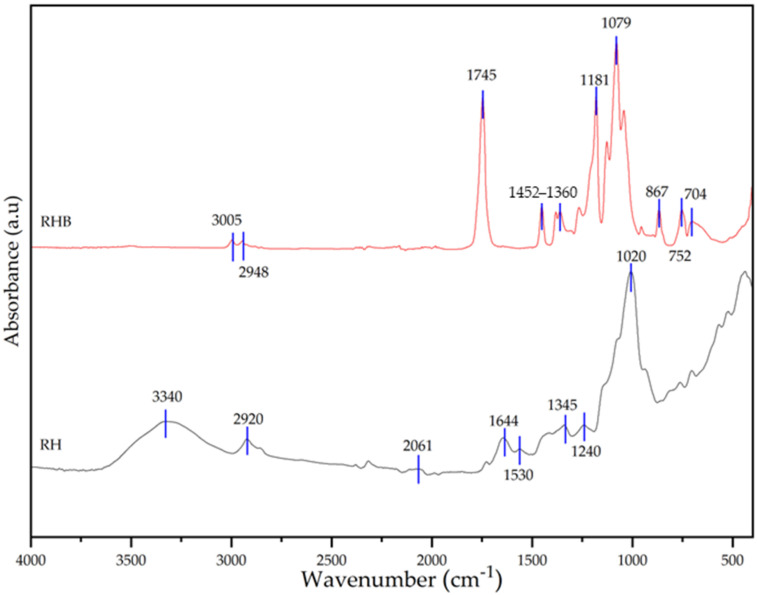
FTIR test on biochar rice husk (RH) and rice husk biochar (RHB).

**Figure 5:**
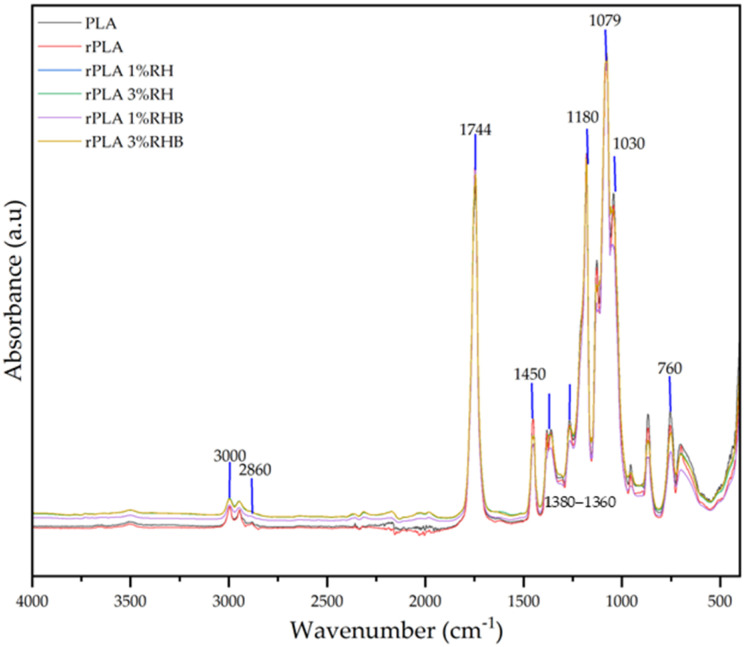
FTIR spectral analysis of PLA, rPLA and rPLA composite films.

FTIR was used to obtain detailed information on the chemical molecular composition of the sample of RH and RHB as well as that of the composite films. The ATR-FTIR spectra of RH and RBH are summarized in Figure 4 and the ATR-FTIR spectra of all composite films are summarized in Figure 4 and Figure 5.

In the ATR-FTIR analysis of uncarbonized rice husk, a notable absorption band at 3332 cm^−1^ is evident, attributed to -OH groups, reflecting hydrogen bonds between hydroxyl groups in the glucose chains of cellulose. This band may also encompass traces of water present in the sample. Vibrational bands in the 2913 cm^−1^, 1507 cm^−1^, and 1369 cm^−1^ regions indicate the presence of C-H bonds, associated with aliphatic organic compounds. Around 2051 cm^−1^, the C=O stretches from carbonyl groups in hemicellulose are observed. C-C group vibrations are found between approximately 1600 cm^−1^ and 1680 cm^−1^, corresponding to lignin. The band around 1027 cm^−1^ is related to the stretching of the acetyl functional group, referred to as C-O. This analysis highlights the main functional groups present in the rice husk, which align with those reported in previous studies on lignocellulosic fibers [34,35,36].

The ATR-FTIR analysis of biochar produced from the carbonization of rice husk reveals significant insights into the chemical transformations and structural modifications that occur during the carbonization process [37]. The characteristic peaks observed in the FTIR spectrum provide valuable information about the functional groups present in the biochar and the extent of changes compared to the uncarbonized rice husk.

The peak observed at 3005 cm^−1^ corresponds to =C-H stretching vibrations, indicating the presence of alkenes or aromatic structures. This suggests that the carbonization process has facilitated the formation of aromatic rings, a key feature of biochar that contributes to its stability and potential applications [38]. The presence of a peak at 2948 cm^−1^ indicates residual C-H stretching from methylene groups, suggesting that while significant transformation has occurred, some aliphatic chains may persist in the biochar matrix. The carbonyl C=O stretching peak at 1745 cm^−1^, although reduced in intensity, suggests that some oxygenated functional groups remain in the biochar, potentially influencing its surface reactivity. Peaks at 1452 cm^−1^ and 1382 cm^−1^ are associated with CH_2_ and CH_3_ bending vibrations, highlighting the presence of methyl and methylene groups that are remnants of the aliphatic structure post-carbonization [39].

The bands at 1265 cm^−1^ and 1181 cm^−1^ indicate C-O stretching vibrations in esters or ethers, suggesting that the carbonization process may preserve some structural elements from the original biomass [40]. The region encompassing 1131 cm^−1^, 1079 cm^−1^, and 1041 cm^−1^ reflects C-O and C-O-C stretching vibrations, consistent with ether linkages or modified cellulose remnants, demonstrating the complexity of the biochar’s internal structure [39]. The band at 966 cm^−1^ may indicate =C-H twisting vibrations, suggesting the presence of unsaturated bonds that could result from incomplete aromatic transformation or new bond formations within the biochar matrix [35]. Finally, peaks at 867 cm^−1^, 752 cm^−1^, and 704 cm^−1^ are typical of out-of-plane bending vibrations in aromatic rings, confirming the development of condensed aromatic structures that are integral to the biochar’s chemical stability and its potential applications [34].

Overall, the FTIR analysis highlights the transformation of rice husk into a more aromatically enriched and structurally stable material through carbonization [40]. The presence of both aromatic and aliphatic features underscores the complexity of biochar and suggests its suitability for a range of functional applications, from soil amendment to use as a filler in polymer composites [41]. This transformation aligns with the existing literature, which describes biochar as a versatile material with significant potential in sustainable technologies [42].

On the other hand, the ATR-FTIR spectra of PLA film reveals several typical distinctive bands that provide insights into the polymer’s chemical structure and interactions. The analysis of these FTIR bands is crucial for understanding the structural integrity and stability of PLA. The peak at 1744 cm^−1^, attributed to the carbonyl stretching vibration of the lactide ester groups, is a prominent feature in PLA spectra [10].

The band at 1450 cm^−1^ corresponds to the bending vibrations of methyl groups (CH_3_) in PLA. The C-O stretching vibrations at 1180 cm^−1^ correspond to ester linkages within PLA [11]. Additionally, the peak at 1079 cm^−1^ reflects C-O-C stretching in glycolic linkages, which are integral to the PLA backbone [2].

The spectral region between 3000 cm^−1^ and 2860 cm^−1^ is characterized by –CH stretching bands. A broad absorption around 3000 cm^−1^, attributed to the cyclohexene group, overlaps with the –CH peaks. The spectra also exhibit the asymmetric stretching of the carbonyl group (C=O) at 1744 cm^−1^, the CH_3_ group at 1450 cm^−1^, and symmetric and asymmetric deformation bands of –CH at 1380 cm^−1^ and 1360 cm^−1^, respectively.

All composite films and rPLA exhibited the characteristic PLA absorption bands. No significant peak shifts or new bands were detected after reprocessing or filler addition, suggesting that the PLA main chain remained chemically unchanged within the detection limits of ATR-FTIR.

### 3.4. Field Emission Scanning Electron Microscopy (FESEM)

The micrographs corresponding to the cross-sectional fracture surfaces obtained by cryo-fracture by previously freezing the sample in liquid N_2_ are shown in Figure 4, where the morphology of each analyzed formulation can be observed.

**Figure 6:**
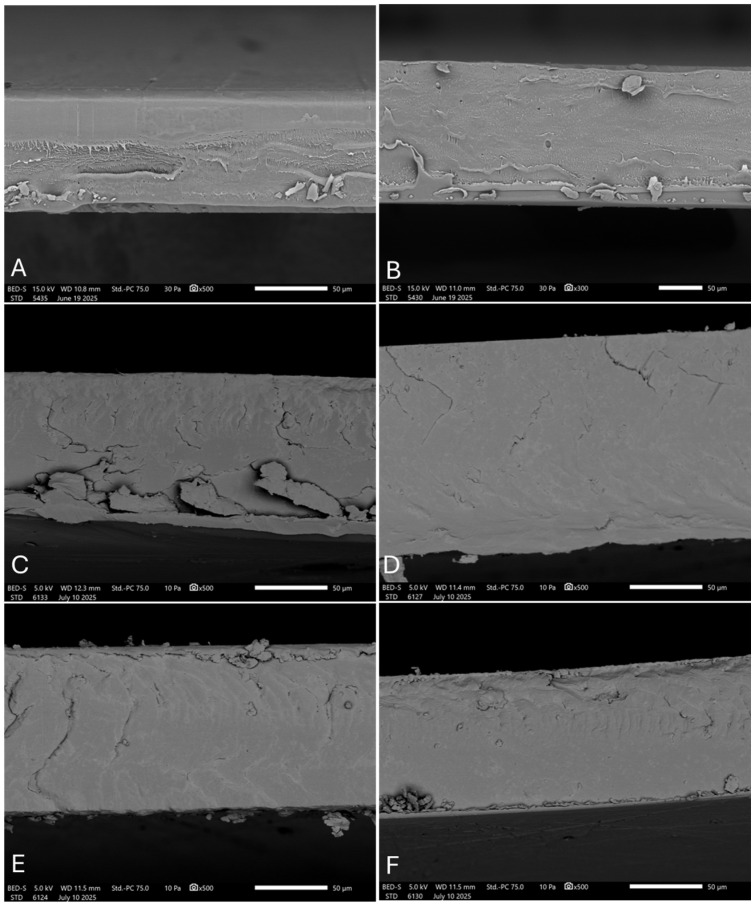
FE-SEM observations at films: (**A**) PLA, (**B**) rPLA, (**C**) rPLA-1%RH, (**D**) rPLA-1%RHB, (**E**) rPLA-3%RH, (**F**) rPLA–3%RHB (white bar = 50 µm).

In the PLA film (Figure 6A), a homogeneous and continuous topography is evident, with a smooth surface and no signs of tearing. This regular morphology is characteristic of brittle fracture with minimal plastic deformation, typical of unmodified semicrystalline PLA matrices, in accordance with previous reports [10]. In contrast, the rPLA film (Figure 6B) exhibits a visibly rougher, yet continuous fracture surface. Unlike PLA (Figure 6A), rPLA (Figure 6B) displays a somewhat more irregular texture attributed to the thermal degradation accumulated during the second reprocessing cycle [10,43]. For the rPLA, and RH- (Figure 6C,E) or RHB (Figure 6D,F)-loaded films, a rougher surface was observed due to the microparticle’s presence, but no reinforcement particles were directly observed embedded within the matrix. Similarly, no cavities, voids, or significant structural defects were identified, suggesting a good particle integration into the polymeric matrix and a reasonably homogeneous distribution, with no apparent morphological alterations compromising the structural integrity of the films [11,17,44]. Therefore, the microstructure showed good interfacial adhesion between the RH or RHB microparticles.

### 3.5. Mechanical Properties

The mechanical properties of PLA and rPLA as well as the rPLA-based composites with rice husk (RH) and rice husk biochar (RHB) provide valuable insights into the impact of natural fillers on polymer matrices. By examining the tensile test properties, Young’s modulus (E_t_), tensile strength (σ_t_), and elongation at break (ɛ_b_), we can understand how these additives influence the structural and functional properties of rPLA-based materials.

**Figure 7:**
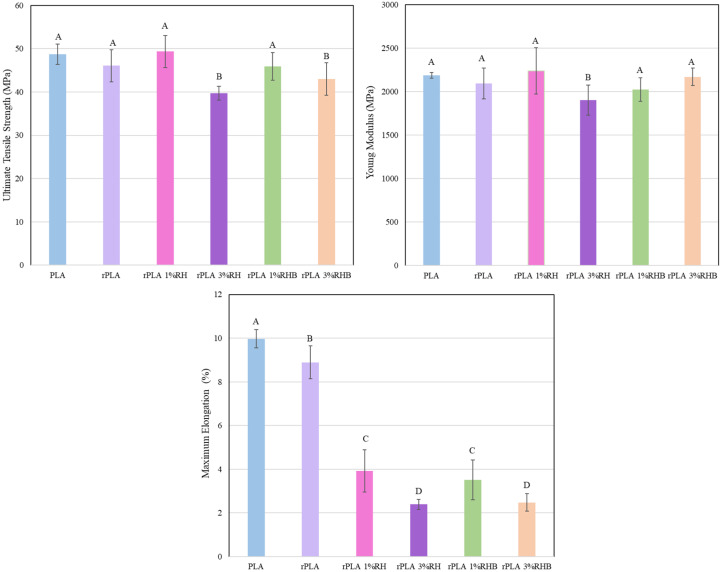
Tensile test properties of PLA, rPLA and rPLA composite films. ^A–D^ Different letters indicate statistically significant differences between formulations *p* < 0.05.

The mechanical properties of PLA and rPLA-based films are summarized in Figure 7. A clear decreasing trend was observed in tensile strength (σₜ), Young’s modulus (Eₜ), and elongation at break (ε_b_) as a result of reprocessing due to the thermal degradation.

The PLA film exhibited the highest mechanical performance, with a tensile strength of 48.7 MPa, a Young’s modulus of 2189.5 MPa, and an elongation at break of 10.0%. Reprocessing reduced these values to 46.1 MPa, 1954.58 MPa, and 8.63%, respectively. This decrease is associated with chain scission during reprocessing, leading to lower molecular weight and reduced chain entanglement [45].

The addition of rice husk (RH) particles further affected the mechanical behavior but in different ways according to the amount and type of rice-husk-based particle. The tensile strength and modulus of rPLA-1%RH did not significantly decrease reaching values of 44.5 MPa and 1944.0 MPa, while elongation dropped significantly to 3.7%, showing a reduction in the flexibility of the films as frequently occurs in polymer composites where the particle limits plastic deformation. Increasing RH content to 3 wt.% exacerbated this effect, with tensile strength and modulus decreasing to 35.3 MPa and 1808.9 MPa, and elongation at break falling to 2.3%. The presence of 3 wt.% of RH introduced rigid, poorly adhered particles in the matrix, acting as stress concentrators and further reducing the deformation capacity [30].

The composites with carbonized rice husk (RHB) showed a more marked reduction in the overall mechanical performance than RH composites. rPLA-1%RHB exhibited a tensile strength of 39.9 MPa, modulus of 1899.1 MPa, and elongation at break of 3%. At 3 wt.% RHB content, these values further decreased to 37.1 MPa, 1850.9 MPa, and 1.7%, respectively, which may indicate limited interfacial adhesion and inefficient stress transfer between the filler and the rPLA matrix.

Overall, the mechanical trend observed here is consistent with the expected response of reprocessed PLA systems containing rigid agro-residue particles. In the present work, reprocessing reduced the tensile strength from 48.7 to 46.1 MPa, the Young’s modulus from 2189.5 to 1954.58 MPa, and the elongation at break from 10.0 to 8.63%, while the incorporation of RH or RHB caused a much stronger reduction in ductility than in stiffness or strength, particularly at 3 wt.%. In this context, Sepúlveda-Carter et al. (2025) reported that reprocessed PLA films exhibited slightly lower tensile strength, Young’s modulus, and elongation at break than virgin PLA, and concluded that the first reprocessing cycles mainly preserve the tensile response despite measurable reductions in viscosity-average molecular weight [10]. Therefore, the decrease observed from PLA to rPLA in the present study is fully consistent with a matrix that has undergone thermo-mechanical degradation but still retains sufficient structural continuity for film formation.

The behavior of RH-filled films also follows the trend widely reported for PLA composites reinforced with untreated lignocellulosic particles. In the present work, 1 wt.% RH preserved tensile strength and modulus to a large extent, but sharply reduced elongation at break, whereas 3 wt.% RH caused a more pronounced reduction in all tensile parameters. This combination of limited stiffening or property retention at low content and marked embrittlement at higher content is consistent with the literature. Lubwama et al. (2025) found that rice husk incorporation into PLA increased the modulus of elasticity but lowered the maximum stress relative to neat PLA [14]. Likewise, Barreto et al. (2024) reported that low amounts of rice husk in PLA filaments strongly decreased elongation at break, in some cases by more than 50%, and attributed the reduction in deformability to stress concentration, voids, and insufficient particle adhesion to the polymer matrix [15]. In this context, the sharp drop in elongation observed here, even when tensile strength remained relatively close to rPLA at 1 wt.% RH, suggests that RH acted mainly as a rigid phase restricting plastic flow rather than as an efficient reinforcing filler. At 3 wt.%, the additional loss of strength and modulus indicates that defect sensitivity and inefficient stress transfer became dominant failure-controlling factors.

A particularly relevant result is that RHB caused a more marked deterioration in tensile properties than RH under the conditions tested. This result is in agreement with some biochar-based PLA systems reported in the literature, where low biochar contents maintained stiffness and, in some cases, tensile strength. Botta et al. (2024), working with recycled PLA filled with poplar biochar, reported an increase in elastic modulus of up to 20% relative to extruded recycled PLA, although elongation at break still decreased [9]. Similarly, Papadopoulou et al. (2025) observed that low biochar from the pyrolysis of pelleted softwood pellet contents in neat PLA slightly enhanced mechanical performance, whereas higher contents reduced it because of lower molecular weight and the detrimental effect of excessive biochar loading [46]. In contrast, Vengadesan et al. (2025) showed that hybrid systems containing treated rice husk and biocarbon could improve strength and ductility when dispersion and interfacial bonding were optimized, but excessive biocarbon loading again led to brittleness and strength loss [16]. Taken together, these comparisons suggest that carbonization alone does not guarantee better reinforcement. In the present films, the RHB particles likely behaved as rigid discontinuities with limited stress-transfer efficiency, so the potential advantages of a more carbonized surface were outweighed by interfacial limitations and filler-induced embrittlement.

### 3.6. Differential Scanning Calorimetry (DSC)

**Figure 8:**
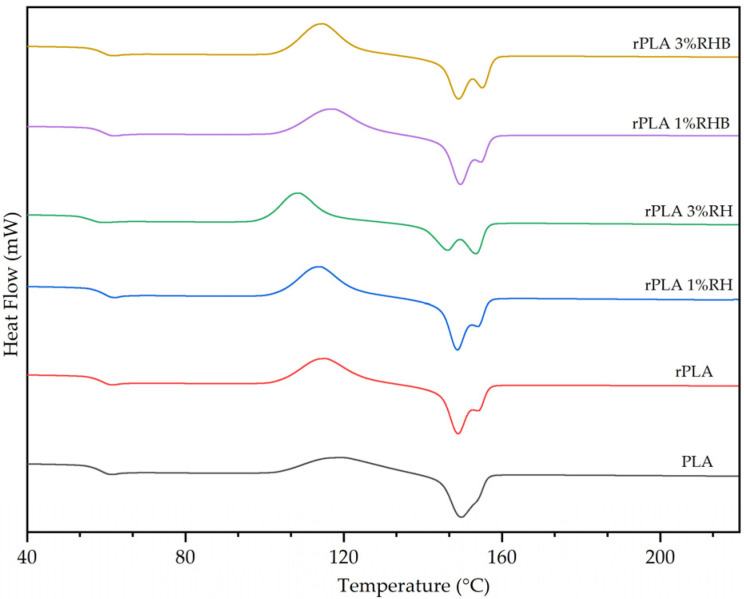
Differential scanning calorimetry (DSC) thermograms obtained during the second heating scan of PLA, rPLA and rPLA composite films.

The DSC thermograms of all films are shown in Figure 8, while the thermal parameters obtained, including the glass transition temperature (T_g_), cold crystallization temperature (T_cc_), melting temperature (T_m_), associated enthalpies (ΔH_cc_ and ΔH_m_), and degree of crystallinity (X_c_) are summarized in Table 1.

The neat PLA film showed the typical behavior of PLA film, whereas the rPLA showed two melting peaks. The multimelting behavior is commonly attributed to the presence of less perfect crystals that initially melt at lower temperatures and subsequently reorganize into more stable and ordered crystalline structures, which remelt at higher temperatures, already observed in mechanically recycled PLA [47].

The T_g_ of the neat PLA films was 59.0 °C and remained practically constant in the reprocessed and composite formulations, with values ranging from 59.2 °C to 59.8 °C. Sepúlveda-Carter et al. reported similar T_g_ values of 58 °C and 58.7 °C for PLA and rPLA, respectively [10]. An exception was observed in the rPLA-3%RH sample, where T_g_ decreased to 55.9 °C. This reduction may be attributed to the higher thermo-mechanical degradation in good agreement with the reduction in the molecular weight of rPLA and the marked increase in MFI values in this formulation, suggesting an increased chain mobility, while the highest content of untreated lignocellulosic fibers generate a less homogeneous matrix and introduce irregularities that favor increased segmental mobility in the amorphous regions of the polymer.

The T_cc_ values progressively decreased from 119.9 °C in neat PLA to 108.5 °C in the rPLA-3%RH composite, indicating an enhancement of the crystallization ability in recycled materials due to the reduction in molecular weight and increased chain mobility. The addition of RH further decreases the cold crystallization temperature. This decrease in T_cc_ in composite films can be associated with a nucleating effect induced both by reprocessing and by the addition of RH particles, promoting molecular rearrangement at lower temperatures and facilitating the crystallization of the rPLA matrix during heating [48], particularly at 3 wt.% loading. The addition of 1 and 3 wt.% of carbonized particles (RHB) exhibited different tendencies. While the incorporation of 1 wt.% of RHB increased the T_cc_ values to 117.3 °C, probably due to the presence of shorter polymer chains that may become partially adsorbed onto the RHB filler surface, which can restrict their mobility and hinder their ability to reorganize into crystalline structures, which requires more thermal energy to crystallize the rPLA matrix. Meanwhile, the addition of a higher content of 3 wt.% of RHB somewhat decreases the Tcc value of rPLA indicating a more homogeneous nucleating effect, probably due to their higher thermal stability of RHB and more regular surface morphology compared to RH [48].

The T_m_ remained stable in most formulations, with values ranging from 148.7 °C to 149.7 °C. However, a significant increase to 153.4 °C was observed in the rPLA-3%RH film sample, suggesting the formation of more stable and ordered crystals. This phenomenon may be related to recrystallization induced by the interaction between rPLA chains and RH particles associated with the formation of more stable crystalline domains, although with a lower total amount of crystals [49]. This increase in T_m_ was not replicated in the RHB formulations, in which the Tm values were mainly maintained, indicating differences in the nucleation mechanism and in the quality of the crystals formed depending on the nature and content of the reinforcement [44].

The melting enthalpy (ΔH_m_) and cold crystallization enthalpy (ΔH_cc_) reflect the amount of energy absorbed and released during the melting and molecular ordering processes and allows to determine the overall crystallinity in the polymeric matrix [50]. The unfilled reprocessed PLA (rPLA) showed a higher crystallinity (X_c_ = 10.0%) compared to neat PLA (X_c_ = 8.0%), which may be attributed to chain redistribution during thermal reprocessing, facilitating spontaneous nucleation during the thermal cycle [51]. The highest ΔH_m_ was recorded for rPLA-3%RHB (44.5 J/g), followed by rPLA-3%RH (41.0 J/g). These values suggest a general improvement in the reprocessed materials’ ability to reorganize their chains and form crystalline regions, compared to PLA (33.4 J/g). Despite exhibiting a lower T_m_, the rPLA-3%RHB formulation showed the highest crystallinity among all samples (X_c_ = 16.3%), indicating an effective nucleating action due to the synergistic effect of shorter polymer chains generated during the polymeric matrix reprocessing and RHB, possibly associated with the porous surface and low hygroscopicity of RHB [52]. In contrast, rPLA-3%RH, although exhibiting a higher T_m_, showed slightly lower crystallinity (X_c_ = 15%), supporting the hypothesis that crystal perfection does not always correlate with the total amount of crystals formed [50,53].

### 3.7. Thermogravimetric Analysis (TGA)

**Figure 9:**
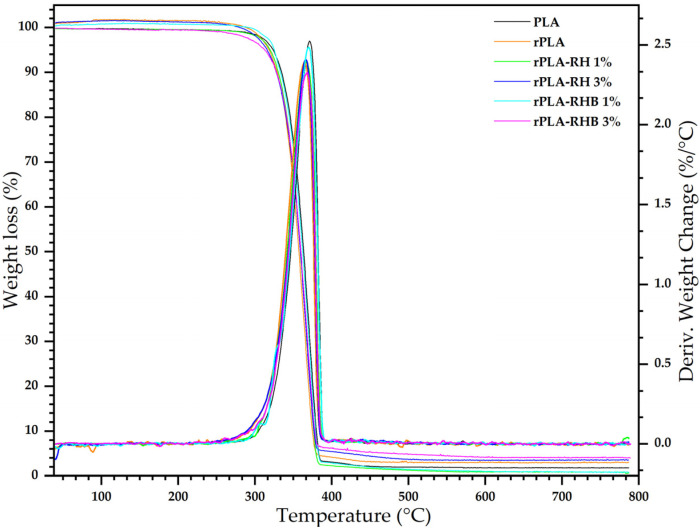
TGA and DTG curves of PLA, rPLA and rPLA composite films.

Figure 9 shows the thermogravimetric curves of all films, while Table 2 compiles the data collected by the thermogravimetric analysis.

PLA films exhibit an initial decomposition temperature of 323.4 °C, indicating good thermal stability for the intended application. The maximum decomposition temperature of 371.2 °C and a maximum mass loss of 65.6% are typical characteristics of PLA, reflecting its standard thermal degradation behavior [8,10,54]. Upon reprocessing, a decrease in the initial decomposition temperature to 318.2 °C and a maximum decomposition temperature of 330 °C is observed in rPLA film, in good accordance with previous studies on mechanically recycled PLA [10,26,55]. This suggests that reprocessing may affect the polymers’ molecular structure, likely due to thermal and mechanical degradation mechanisms. The increase in maximum mass loss to 66% could be attributed to the greater release of volatile degradation products generated during reprocessing [10,26].

The addition of 1 wt.% of rice husk (RH) to rPLA results in a slight decrease in the initial decomposition temperature to 319.4 °C and in the maximum decomposition temperature to 366.4 °C. The reduction in maximum mass loss to 66% may indicate some level of interaction between the PLA matrix and the filler, potentially improving resistance to thermal decomposition by limiting the extent of mass loss [1,50]. When increasing the RH content to 3 wt.%, a further reduction in both the initial (315.7 °C) and maximum (366.2 °C) decomposition temperatures is observed. This trend suggests that higher RH content may serve as degradation initiation sites due to their complex chemical nature. However, the lower maximum mass loss of 57.6% implies that RH might also contribute to some degree of thermal stabilization, possibly through char formation or barrier effects [2,56,57].

The incorporation of 1 wt.% of carbonized rice husk (RHB) yields an initial decomposition temperature of 325.2 °C, slightly higher than that of rPLA and rPLA-RH, and a reduced maximum decomposition temperature of 369.6 °C. The maximum mass loss remains at 65.7%, indicating that while carbonized RH does not significantly impact mass loss compared to RH, it may alter the degradation pathway [31]. When the RHB content is increased to 3 wt.%, the initial decomposition temperature decreases to 312.7 °C, and the maximum decomposition temperature drops further to 368.2 °C. Despite this decrease in thermal stability, the maximum mass loss remains at 66.1%, similar to that of the non-carbonized RH system. This suggests that carbonized husk affects the onset of degradation more markedly but does not significantly influence the final degradation extent [52].

### 3.8. Water Contact Angle

**Figure 10:**
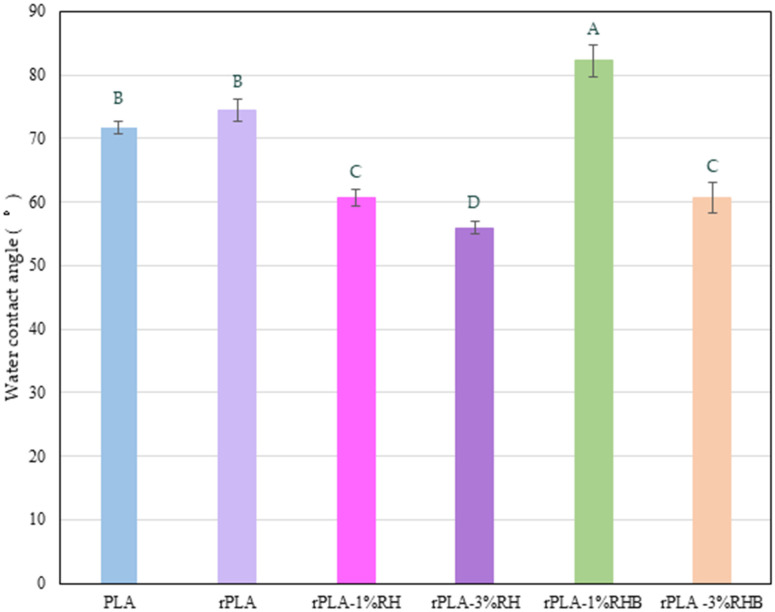
Static water contact angle (°) of PLA, rPLA and rPLA composite films. ^A–D^ Different letters indicate statistically significant differences between formulations, *p* < 0.05.

The surface wettability of the developed films was evaluated through static water contact angle (WCA) measurements. The results (Figure 10) revealed clear variations in surface hydrophilicity as a function of the polymer matrix type and the nature of the added fillers.

The PLA exhibited an average WCA of 71.8 ± 1.0°, which falls within the characteristic range reported for untreated PLA films (60–80°) [10,58]. This value indicates moderate hydrophilic behavior, attributed to the presence of polar carbonyl and ester groups capable of forming hydrogen bonds with water molecules [10]. The slight variability in the values reported by different authors has been associated with differences in crystallinity, surface roughness, and molecular orientation [10,32].

After reprocessing (rPLA), the WCA of rPLA film increased to 74.5 ± 1.7°, suggesting a reduction in wettability and a somewhat increase in surface hydrophobicity. This effect can be explained by molecular rearrangements and increased surface crystallinity induced during thermal reprocessing, which reduces the exposure of polar groups [32].

The incorporation of lignocellulosic rice husk (RH) particles led to a marked decrease in contact angle, with values of 60.7 ± 1.4° for 1 wt.% and 56.0 ± 0.9° for 3 wt.%. This trend indicates a progressive increase in surface hydrophilicity, attributed to the polar nature of rice husk, which is rich in hydroxyl and carboxyl groups that favor water interaction [17]. Moreover, the addition of RH particles increases surface roughness and interfacial heterogeneity, promoting water spreading [59]. Similar trends have been reported in PLA-based biocomposites reinforced with plant fibers or lignocellulosic nanoparticles, where the incorporation of hydrophilic materials decreases the WCA and enhances surface wettability [17].

In contrast, the addition of rice husk biochar (RHB) at 1 wt.% loading levels resulted in a significant increase in the contact angle (82.3 ± 2.5°), reflecting a more hydrophobic surface. This behavior is attributed to the carbonaceous nature and low polarity of biochar, since carbonization removes most of the hydrophilic components of biomass (cellulose, hemicellulose) and generates an aromatic surface with few oxygenated groups [46]. These characteristics reduce the surface energy and consequently the affinity of water for the rPLA-RHB-based film surface. Comparable results have been reported in PLA composites containing biochar or activated carbon, in which the contact angle can exceed 80° [46,52]. However, increasing the biochar content to 3 wt.% caused an abrupt decrease in WCA to 60.8 ± 2.4°, suggesting an exposure of polar groups at the film surface. At higher concentrations, biochar agglomerates can introduce topographical irregularities and surface defects that enhance local water retention and reduce the water contact angle value [11,17,44]. Additionally, this result is in good agreement with the reduction in MFI value in this formulation that suggests thermal degradation. The formation of additional polar end groups (–COOH and –OH) associated with degradation, together with increased surface roughness caused by RHB agglomeration, promotes a surface wettability increment, despite the intrinsically hydrophobic character of biochar. This nonlinear behavior confirms that the hydrophobicity induced by carbon-based fillers strongly depends on their dispersion, loading, and surface functionalization [46].

Overall, the results demonstrate that the wettability of rPLA films can be tuned through the incorporation of natural or carbonized reinforcements, controlling the balance between chemical polarity and surface topography. The formulations containing RH exhibited more hydrophilic surfaces, suitable for applications requiring adhesion or compatibility with polar matrices, whereas the rPLA-1%RHB formulation showed enhanced hydrophobicity, which may be advantageous for packaging or water-resistant material applications [60].

### 3.9. Water Absorption

**Figure 11:**
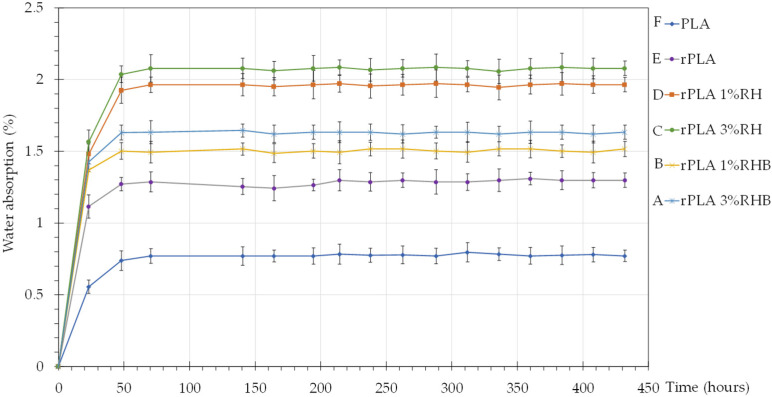
Water absorption of PLA, rPLA and rPLA composite films. ^A–F^ Different letters indicate statistically significant differences between formulations, *p* < 0.05.

Figure 11 presents the results of the water absorption test, which provides crucial information about the materials’ ability to retain moisture under specific conditions. These results are essential for evaluating the suitability of the composites in applications where water exposure is a determining factor.

PLA exhibits the lowest water absorption rates throughout the test duration, indicating its inherent hydrophobic properties. The absorption rate stabilizes around 0.77% after 70.5 h, showing very limited increase thereafter. rPLA film shows slightly higher water absorption rates than neat PLA, which might be due to structural compromises caused by the recycling process, making it slightly more susceptible to moisture uptake. These results were similar to those reported in previous studies by [44,61,62].

Both rPLA-1%RH and rPLA-3%RH composites show a progressively higher water absorption rate compared to their rPLA counterpart; with 3 wt.% RH content the composite shows the highest absorption, reaching up to around 2.08%. This increase is likely due to the hydrophilic nature of cellulose found in rice husks, which absorbs water more readily than the PLA matrix [61,63].

rPLA with 1 wt.% and 3 wt.% of RHB also demonstrated higher water absorption rates than neat PLA and rPLA films, but generally, the rates are slightly lower than those of the RH composites. This could be attributed to the more hydrophobic nature, produced by the carbonization process of the rice husk, which helps reduce the overall moisture affinity of the composite [52].

The water absorption ranking observed in the present study, namely PLA < rPLA < RH-filled composites, with RHB-filled films showing an intermediate behavior, is fully consistent with the behavior commonly reported for PLA-based composites containing lignocellulosic fillers. In our results, neat PLA exhibited the lowest equilibrium water uptake, RH-containing composites showed the highest absorption, and RHB-containing composites absorbed less water than RH-filled films but still more than neat PLA and rPLA. This trend is consistent with the current understanding that natural lignocellulosic fillers increase water absorption because their hydroxyl-rich structure promotes both direct sorption and capillary transport through the filler–matrix interphase. Azka et al. (2024) [64], in a recent review focused specifically on natural-fiber-reinforced PLA composites, emphasized that water uptake in these systems is governed not only by the intrinsic hydrophilicity of the fiber, but also by interfacial cavities and diffusion pathways generated during processing. Similarly, Singh et al., (2022) [65] reported that water absorption in PLA biocomposites increased with rice husk and wood-derived filler loading, rising from 0.36% in unfilled PLA to 1.92% in filled systems, which they attributed to the water-binding and swelling ability of lignocellulosic constituents such as cellulose, hemicellulose, and lignin.

The slightly higher water absorption observed for rPLA relative to neat PLA can also be rationalized in light of previous studies on reprocessed PLA systems. Although the increase in our work was moderate, it is consistent with the general effect of thermo-mechanical reprocessing, which tends to promote chain scission, generate more chain ends, and introduce microstructural discontinuities that facilitate moisture ingress. In this regard, Gil-Castell et al. (2022) [66] reported that reprocessed plasticized PLA bionanocomposites showed higher absorbed water and solubility coefficients than neat PLA, and they associated this behavior with the combined contribution of matrix degradation, filler-related heterogeneity, and the formation of micro- and nanovoids in the interphase. Even though their system involved nanofibrillated cellulose and plasticization, the mechanistic interpretation remains relevant here, since it supports the view that processing history itself can make PLA-based matrices more susceptible to water penetration when compared with their virgin counterparts.

The comparatively lower water absorption of RHB-filled films with respect to RH-filled films is also in good agreement with recent literature on carbonized biomass fillers. Vengadesan et al. (2025) [16] showed that increasing the fraction of biocarbon in PLA/rice-husk-based composites reduced water absorption because carbonization decreases the number of hydroxyl groups available for water sorption and improves the moisture resistance of the filler phase. Their study also noted, however, that excessive biocarbon loading may induce particle aggregation and structural heterogeneity, which can compromise composite performance.

Therefore, the present results suggest that carbonization of rice husk partially suppressed the strong hydrophilic contribution of the untreated RH, thereby lowering water uptake, but did not fully eliminate moisture transport because the RHB particles could still contribute porosity, interfacial discontinuities, and localized water retention sites. This interpretation is particularly consistent with the fact that the RHB containing composites in our study remained more absorbent than neat PLA and rPLA, while still performing better than the corresponding RH containing formulations.

### 3.10. Water Vapor Transmission Rate

**Figure 12:**
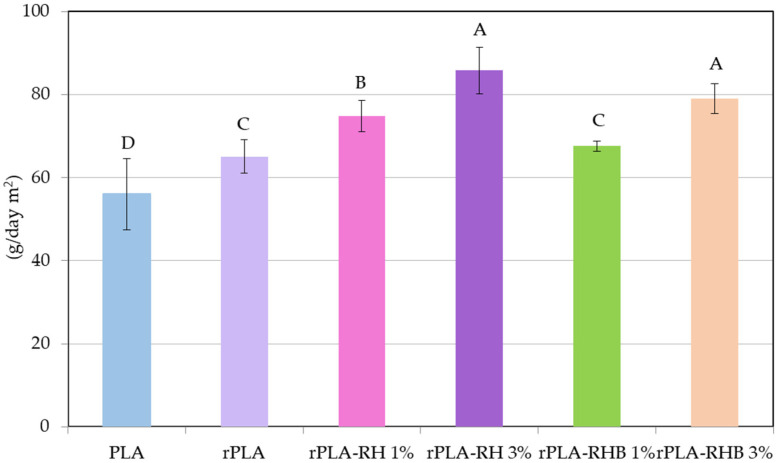
Water vapor transmission rate (WVTR) of PLA, rPLA and rPLA composite films. ^A–D^ Different letters indicate statistically significant differences between formulations, *p* < 0.05.

Figure 12 presents the results from the water vapor transmission rate (WVTR) test, which evaluates the permeability of the PLA and rPLA films and their composites with natural fillers, rice husk (RH) and rice husk biochar (RHB).

PLA exhibits the lowest WVTR of 56.1 gr day^−1^ m^−2^, in accordance with its hydrophobic polymer matrix and with already reported values for neat PLA [10]. rPLA film shows a higher WVTR at 65.1 gr day^−1^ m^−2^ compared to neat PLA, because the recycling process slightly degraded the polymeric matrix reaching in shorter polymer chains that increased the polymers’ chain mobility, increasing its permeability to water vapor. This result is in good agreement with the reduction in the molecular weight and the marked increase in MFI values in rPLA. The increased chain mobility allows water molecules to more easily permeate the rPLA polymeric matrix than the PLA one. A similar effect of reprocessing on moisture barrier performance has been reported for recycled PLA, where molecular degradation during processing led to reduced barrier efficiency, while Sepúlveda-Carter et al. (2025) observed that simulated industrial reprocessing of PLA caused a slight increase in WVTR, attributed to changes in chain architecture and transport pathways [10].

The rPLA-1%RH further increased the WVTR value to 74.8 gr day^−1^ m^−2^. This indicates that the addition of RH with a hydrophilic nature facilitates the water interaction and further reduces the water barrier properties, allowing more water vapor to pass through. Accordingly, rPLA-3%RH shows a significant rise in WVTR to 85.8 gr day^−1^ m^−2^, the highest among the samples tested. rPLA-1%RHB exhibits a WVTR of 67.6 gr day^−1^ m^−2^, which is slightly higher than rPLA but much lower compared to RH composites. This suggests that RHB, due to its possibly more carbonized and porous structure, might interact differently with the PLA matrix, leading to a less pronounced increase in permeability. Similarly, rPLA-3%RHB records a WVTR of gr day^−1^ m^−2^. Although this is lower than rPLA with 3 wt.% RH, it is still substantially higher than the neat PLA and rPLA, indicating that higher concentrations of RHB also compromise the barrier properties but to a lesser extent than RH.

The trend observed in the present study, namely the increase in WVTR after reprocessing and the further deterioration caused by agro-residue incorporation, is consistent with previous reports on recycled and filler-modified PLA systems. In particular, the ranking described here, with the lowest WVTR for neat PLA, a higher permeability for rPLA, a strong additional increase for RH-filled films, and a comparatively less severe increase for RHB-filled films, follows the same mechanistic framework generally reported for PLA barrier behavior, in which chain scission, higher amorphous-phase mobility, and interfacial heterogeneity facilitate water vapor diffusion through the matrix [10,67,68].

The more pronounced rise in WVTR for RH-containing formulations is also in agreement with the behavior commonly described for PLA composites reinforced with untreated lignocellulosic fillers. Lubwama et al. (2025) highlighted that the incorporation of rice husk microparticles into PLA is associated with increased water vapor permeability [14], while Marano et al. (2022) noted that natural fillers may only improve barrier performance at low contents and under good dispersion conditions [68]. Otherwise, their hydrophilic character, agglomeration tendency, and poor interfacial adhesion can generate preferential diffusion pathways and microvoids that increase permeability [14,68]. This interpretation is further supported by previous studies on mechanically recycled PLA reinforced with lignocellulosic or cellulosic particles. Beltrán et al. (2020) reported that hydrophilic yerba-mate nanoparticles increased the WVTR in mechanically recycled PLA [54]. Meanwhile, Agüero et al. (2023) found that the incorporation of microbial cellulose into plasticized mechanically recycled PLA also increased the WVTR because the filler phase promoted moisture transport through the film structure [11]. Therefore, the marked increase from rPLA to rPLA-1%RH and especially to rPLA-3%RH suggests that, in the present system, the hydrophilic nature of RH and the formation of interfacial defects dominated over any potential tortuosity effect [14].

By contrast, the lower permeability penalty observed for RHB-filled films relative to RH-filled films suggests that carbonization partially reduced sorption-driven transport, likely because the filler became less polar than the untreated husk. However, the fact that RHB did not reduce the WVTR below that of rPLA indicates that this potential advantage was counterbalanced by other structural factors, such as filler porosity, incomplete matrix encapsulation, and local aggregation at higher loading. This type of competing behavior has already been observed for PLA/biochar systems, in which barrier performance depends not only on the lower polarity of the carbonized filler, but also on its surface chemistry, particle size, and dispersion quality within the matrix [52,68].

### 3.11. Disintegration Under Composting Conditions

**Figure 13:**
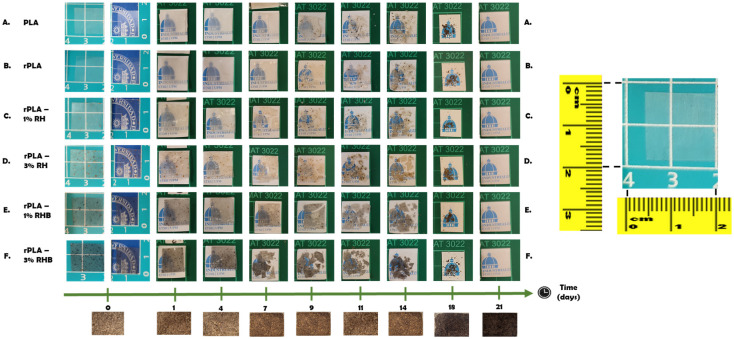
Evolution of the visual properties of PLA, rPLA and rPLA composite films during compost disintegration.

**Figure 14:**
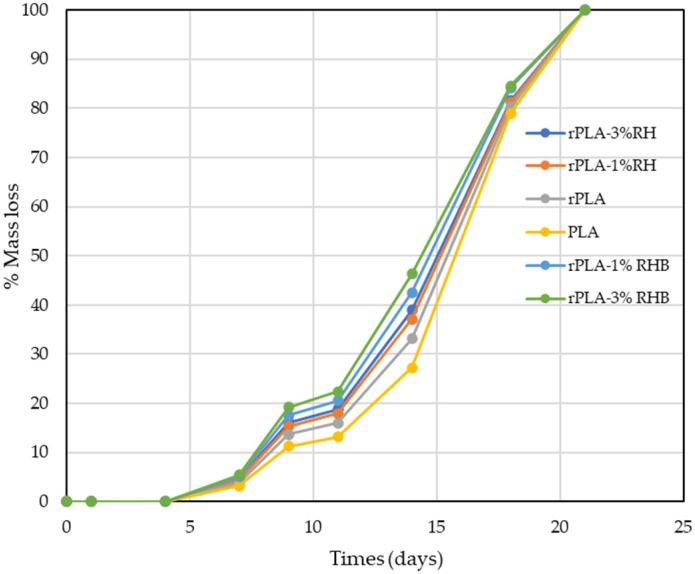
Disintegration curves under composting conditions.

The disintegration ability of the composite materials under controlled composting conditions at a laboratory scale level was assessed based on the mass loss over time while buried. The visual changes in the unearthed film samples and the solid compost across various incubation periods are depicted in Figure 13, whereas the mass loss induced by the composting duration for all samples is presented in Figure 14.

From the first day of the experiment, the PLA films showed a noticeable decrease in transparency, which is indicative of the early onset of hydrolytic reactions and microbial activity facilitated by the elevated temperature (58 °C) and high moisture content of the composting medium [17]. This rapid loss of transparency can be attributed to the water absorption by hydrophilic components within the film, possibly exacerbated by the presence of natural fillers like rice husk and biochar, which increases the hydrophilicity of the composite. As the degradation progressed, the films became increasingly opaque. This loss of transparency is typically associated with the physical and chemical breakdown of the PLA polymeric matrix, where the structural integrity of the films is compromised, and the material starts to exhibit visible signs of enzymatic breakdown due to microbial colonization [18]. By day 21, the films had completely disintegrated and integrated into the composting substrate. At this stage, the films lost all transparency as they broke down into smaller fragments or dissolved at the molecular level, effectively merging with the organic matter in the compost [17].

Samples containing 3 wt.% of RH or RHB exhibited higher mass loss throughout composting than the corresponding 1 wt.% formulations, indicating that increasing filler content accelerated the disintegration of the rPLA films. In the present study, rPLA-3%RHB reached 46.4% mass loss on day 14 and 84.6% on day 18, whereas rPLA-3%RH reached 38.9% and 81.7%, respectively; similarly, rPLA-1%RHB outperformed rPLA-1%RH at the same time points, while neat PLA and rPLA showed the slowest intermediate-stage disintegration. This behavior is in good agreement with previous reports on PLA-based biocomposites reinforced with lignocellulosic fillers, where the incorporation of hydrophilic natural particles promotes water uptake, facilitates hydrolytic chain scission, and generates interfacial discontinuities that accelerate fragmentation.

Cabrera-García et al. reported greater mass loss and faster sample deterioration in disintegration tests for natural-fiber-reinforced PLA than for neat PLA [69], while Scaffaro et al. (2025) recently emphasized that cellulose-containing biodegradable composites generally show faster compost disintegration because the filler phase enhances hydrolytic and enzymatic attack [70]. In addition, the slightly faster disintegration of rPLA compared with neat PLA is consistent with the findings of O’Loughlin et al. (2026), who observed that mechanically recycled PLA exhibits a modest increase in compost disintegration rate due to processing-induced molecular degradation [71]. The fact that all formulations reached full disintegration by day 21 is also consistent with previous work on thin PLA-based materials under controlled composting conditions, including reprocessed PLA systems that fully disintegrated in less than three weeks, and PLA films reported to degrade within approximately one month under simulated industrial composting conditions [10].

The slightly faster disintegration observed for RHB-filled films relative to RH-filled films is particularly noteworthy. Although carbonization generally makes rice-husk-derived fillers less polar, our own results already indicate the comparatively less polar nature of RHB, especially at low loading, and the present composting data suggest that the porous architecture and higher internal surface area generated during pyrolysis may have played a more dominant role than surface polarity alone. Indeed, recent work on rice husk/biocarbon-filled PLA systems has shown that biocarbon develops a more pronounced microporous structure and larger internal surface area than raw rice husk, which can favor moisture ingress and local interfacial heterogeneity. Moreover, recent composting studies indicate that biochar can actively accelerate PLA degradation by modifying the local physicochemical environment and microbial processes during composting. This interpretation is especially relevant here because higher crystallinity is usually associated with slower PLA hydrolysis under composting conditions. Therefore, the faster mass loss of the RHB-containing films suggests that porosity, defect generation, and easier compost-medium penetration outweighed the crystallinity-related resistance to degradation during the early and intermediate stages of disintegration [16,72,73].

## 4. Conclusions

Although PLA is bio-based, it is considered plastic under Regulation (EU) 2025/40 and is therefore subject to mandatory recycled content requirements for packaging from 2030. In this context, this study simulated the valorization of industrial PLA waste streams, such as defective parts, trimming edges, burrs, and production scraps, by mechanically reprocessing neat PLA through two extrusion cycles and subsequently incorporating rice husk (RH) and rice husk biochar (RHB) as agro-residue fillers. The results demonstrated that both particles modified the processing–structure–property relationship of mechanically reprocessed PLA (rPLA) films.

Reprocessing increased melt flow and reduced intrinsic viscosity and viscosity-average molecular weight, confirming chain scission and lower melt viscosity after repeated thermal processing. The incorporation of RH moderately reduced melt flow, whereas RHB produced a concentration-dependent response, suggesting distinct filler–matrix interactions during melt processing. Tensile testing showed that the main mechanical consequence of both fillers was embrittlement, as reflected by the marked decrease in elongation at break (≈1.7–3.7%) compared with neat PLA (~10%). At low filler loading (1 wt.%), stiffness and tensile strength were generally maintained or only moderately affected; however, the strong loss of ductility at 3 wt.% indicates that fracture increasingly became governed by stress concentration, interfacial limitations, and defect sensitivity in filled rPLA films.

Thermal analysis further showed that RH, and especially RHB, promoted crystallization and increased the degree of crystallinity (up to ~16%), which likely contributed to the reduced strain-to-failure observed in the composites. Surface and barrier-related behavior were also tunable according to filler type: RH increased hydrophilicity and moisture sensitivity, whereas low RHB loading (1 wt.%) improved hydrophobicity. Despite these differences, all formulations reached complete disintegration under composting conditions within 21 days, confirming that the incorporation of these agro-residues did not suppress the compostable character of the films under the tested conditions.

From an application standpoint, these findings suggest that reprocessed PLA films filled with RH or RHB could be considered for short-service-life applications with the additional advantage of being compostable, particularly where full structural performance is not the primary requirement. Potential uses include single-use packaging components, disposable liners, secondary food-contact packaging with moderate mechanical demand, and agricultural or mulch-film applications. Among the evaluated formulations, low RHB contents appear especially promising for applications requiring somewhat improved moisture resistance while maintaining compost disintegration behavior.

Overall, the results highlight that the successful development of rPLA-containing rice husk residue films depends not only on incorporating bio-based fillers, but also on the low amount for controlling interfacial compatibility and dispersion to mitigate embrittlement while preserving the circular economy benefits that make these systems attractive for short-lived packaging and agricultural applications.

## Figures and Tables

**Table 1:** The thermal properties of poly(lactic acid) (PLA) and PLA composites reprocessed (rPLA) with rice husk (RH) and carbonized rice husk (RHB) were evaluated, including measurements of glass transition temperature (T_g_), cold crystallization temperature (T_CC_), melting temperature (T_m_), cold crystallization enthalpy (ΔH_CC_), enthalpy of fusion (ΔH_m_), and degree of crystallinity (X_c_).

Sample (Film)	T_g_ (°C)	T_cc_ (°C)	T_m_ (°C)	ΔH_CC_ (J/g)	ΔH_m_ (J/g)	X_c_ (%)
PLA	59.0	119.9	149.7	26.	33.4	8.0
rPLA	59.2	115.2	148.9	29.1	38.4	10.0
rPLA 1%RH	59.7	114.1	148.7	29.2	35.4	6.7
rPLA 3%RH	55.9	108.5	153.4	27.5	41.0	15.0
rPLA 1%RHB	59.8	117.3	149.4	28.5	36.1	8.3
rPLA 3%RHB	59.2	114.5	149.0	29.8	44.5	16.3

**Table 2:** Comparison of decomposition temperatures and mass loss in different PLA film samples.

Sample	Initial Decomposition Temperature (°C)	Maximum Decomposition Temperature (°C)	Maximum Mass Loss Percentage
PLA	323.4	371.2	65.6
rPLA	318.2	364.7	66.2
rPLA-RH 1%	319.4	366.4	66.0
rPLA-RH 3%	315.7	366.2	57.6
rPLA-RHB 1%	325.2	369.6	65.7
rPLA-RHB 3%	312.7	368.2	66.1

## Data Availability

The original contributions presented in this study are included in the article. Further inquiries can be directed to the corresponding authors.
